# Effects of a positive psychological intervention on psychological states and functional brain imaging of nurses in transition training: a quasi-experimental study

**DOI:** 10.3389/fnhum.2025.1492298

**Published:** 2025-06-24

**Authors:** Li He, Haiyan Wang, Zhiwei Guo, Yu Zhou, Lu Hu, Juan Zhao, Yi Chen, Li Xiang

**Affiliations:** ^1^Department of Nursing, Beijing Anzhen Nanchong Hospital of Capital Medical University & Nanchong Central Hospital, Nanchong, China; ^2^Institute of Brain Function, Department of Radiology, Beijing Anzhen Nanchong Hospital of Capital Medical University & Nanchong Central Hospital, Nanchong, China

**Keywords:** anxiety, depression, nurses, psychological intervention, functional brain imaging

## Abstract

**Objective:**

To explore the effects of a positive psychological intervention on the psychological state and brain function of trained nurses during the transition period.

**Methods:**

Randomization was used to assign 130 trained nurses to the observation and control groups. The control group underwent routine training, while the observation group underwent a 12-week positive psychological intervention based on the “Three Good Things” program. The Core Self-Evaluation Scale, Patient Health Questionnaire Depressive Symptom Cluster Scale, Generalized Anxiety Disorder Scale, and resting-state functional magnetic resonance imaging were used for data collection pre- and post-intervention. The amplitude of low-frequency fluctuation, fractional amplitude of low-frequency fluctuation, and regional heterogeneity indices were calculated to assess the functional brain activity of the participants. Pearson’s correlation coefficient was used to assess the relationship between the changes in brain function and behavior.

**Results:**

There was no difference in the general information of the two groups pre-intervention. Post-intervention, the observation group reported higher self-evaluation scores and lower anxiety and depression scores than the control group. The amplitudes of low-frequency fluctuation of the observation group were significantly higher than those of the control group in the bilateral calcarine gyrus, left cuneus lobe, left supramarginal gyrus, left middle temporal gyrus, left inferior frontal gyrus, left inferior parietal lobe, right superior parietal lobe, right angular gyrus, and right middle temporal gyrus. The observation group displayed significantly higher regional heterogeneity values in the bilateral superior parietal lobe, bilateral supramarginal gyrus, and bilateral inferior parietal lobe (*p* < 0.05).

**Conclusion:**

Positive psychological interventions based on the “Three Good Things” program can assist nurses in training during the transition period to enhance their core self-assessment ability, alleviate their anxiety and depression, and improve their sense of occupational identity and mental health.

## 1 Introduction

Standardized training is a transitional stage in which nurses gain competency in clinical work and face many role changes. In this process, nurses need to enter a new department to understand the complexity of the patient’s condition. Here, their nursing skills are not trained, but rather, their lack of interpersonal communication skills, and other aspects of the training (Sun et al., 2018), result in physiological, psychological, knowledge and skills, social, cultural, and professional development, and cause disorientation, confusion, uncertainty, and suspicion of the position, which is referred to as transition conflict (Liao et al., 2023; Zhu et al., 2023). The “Three Good Things” positive psychological intervention is a new model of psychological intervention developed by Seligman (Craig Phillips and Kenny, 2015), based on the theory of positive psychology, which aims to help people form optimistic, confident, and strong attitudes, improving their quality of life and work. The study found that trained nurses are not optimistic about the career status of nursing and report low self-core evaluation scores. Self-core evaluation is negatively correlated with the level of transition shock, which affects the stability of the nursing team and the quality of nursing care if they cannot cope well with the clinical transition to trained nurses (Zamanzadeh et al., 2015). Therefore, helping trained nurses to transition smoothly and adapt to their new roles as soon as possible has become the primary concern of global nursing human resource management. This study aimed to investigate the effects of the “Three Good Things” intervention on the psychological state, core self-evaluation, and neurological activity imaging performance of trained nurses during the role transition period to provide a base of knowledge for the improvement of clinical interventions and psychological health of nurses in the role transition period.

## 2 Methods

### 2.1 Study participants

Using the whole-group sampling method, 130 newly enrolled nurses from a hospital were selected as study subjects. The inclusion criterion was informed consent to participate. The exclusion criteria were as follows: (1) Recently accepted for other similar studies; (2) Severe psychiatric disorders, such as schizophrenia and major depression; (3) Relative and absolute contraindications to MRI, such as cardiac pacemakers; and (4) Severe visual and auditory disorders or other systemic disorders. Dropout criteria were those who stopped the protocol midway, did not complete the intervention, or those whose images were unsatisfactory. The study participants were randomly divided into a control group (*n* = 65) and an observation group (*n* = 65). To avoid contamination of the intervention program due to contact communication between the observation and control groups, the study was grouped according to the ward. In this study, all hospital wards were divided into two types of groups: internal medicine and acute and critical care wards, surgery and other wards (including operating room, oncology, geriatrics, pediatrics, etc.). Using the coin-flip method, with the coin-flip being done independently by third-party personnel who were not involved in the design and implementation of the study. When the coin was heads up, nurses from the internal medicine and acute and critical care wards were used as the observation group, and nurses from the surgical and other wards as the control group; When the coin is facing up, the nurses of internal medicine and acute and critical care wards become the control group, and the nurses of surgery and other wards are the observation group. After the grouping is completed, all the research subjects are registered with a unified number to ensure that the grouping process is traceable and verifiable.

### 2.2 Intervention methods

The routine practice control group (hereinafter referred to as control group), which included pre-service training and entry training, specifically on nurses’ career planning, interpersonal communication, and nurse–patient communication skills, coping with stress during the role transition period, analysis of core nursing systems, nursing-related laws and regulations, nursing etiquette, departmental rules and regulations, and specialty knowledge training. Trained nurses were encouraged to ask teachers or administrators for advice in the WeChat group or through private messages when they encountered problems. Administrators regularly provided mental health knowledge in the WeChat group with the guidance of psychologists.

The observation group received a positive psychological intervention based on the control group. The research team combined clinical experience, literature research, expert consultation, and other strategies to initially build the intervention, with the specific measures as follows: The intervention lasted 12 weeks, starting in August 2021, and comprised training on positive psychology knowledge through lectures, classroom interactions, and off-campus learning in week 1, including topics such as positive mindset for a happy life; positive cognition; and emotion management from a positive psychology perspective. In weeks 2–4, the nurses underwent self-study on relevant positive psychology knowledge. Additionally, a WeChat group was established, where the administrator supervised and guided the nurses to check in regularly and record “Three Good Things”. In weeks 5–12, nurses targeted learning about positive psychology according to their own situation, they were urged to maintain daily records, and each person was asked to share “Three Good Things” once a week. The administrator was to pay attention to the response and lead the nurses to reflect on the reasons why the good things happen.

## 3 Article types

This was a quasi-experimental study. The study was granted ethical approval by the Hospital Ethics Committee (No.2021–088).

## 4 Evaluation indicators

### 4.1 General information questionnaire

This questionnaire included gender, education, title, marital status, residence, nursing experience (year) and family health status, and was self-reported by participants.

### 4.2 Core self-evaluations scale (CSES)

This one-dimensional self-assessment scale consists of 10 items. A five-point scale was used, with scores ranging from 1 (completely disagree) to 5 (completely agree). Items 2, 3, 5, 7, 8, and 10 were reverse scored. The total score ranges from 10 to 50, with higher scores indicating higher levels of core self-evaluation. Cronbach’s alpha for the total scale was 0.83.

### 4.3 Patient health questionnaire depressive symptom cluster scale (PHQ-9)

This scale consists of nine items that assess the frequency of depressive symptoms over the past 2 weeks. Each entry is rated on a 4-point scale ranging from 0 (not at all) to 3 (almost daily). The total score ranges from 0–27, with 0–4 indicating no depression, 5–9 indicating mild depression, 10–14 indicating moderate depression, 15–19 indicating moderately severe depression, and 20–27 indicating severe depression. Cronbach’s alpha coefficient for the scale was 0.901.

### 4.4 Generalized anxiety scale (7-item generalized anxiety disorder scale, GAD-7)

This scale consists of seven entries rated on a 4-point scale ranging from 0 (not at all) to 3 (almost every day). The total score is 21, with 0–4 indicating no anxiety, 5–9 indicating mild anxiety, 10–14 indicating moderate anxiety, and ≥15 indicating severe anxiety. The Cronbach′s alpha coefficient for the scale was 0.918.

### 4.5 Cranial MRI

#### 4.5.1 MRI

An 8-channel head coil of a 1.5 T MRI scanner (GE Signa HDxt, GE Healthcare, Milwaukee, USA) was used. Scanned images included T1WI, T2WI, T2-FLAIR, BOLD fMRI, and 3D high-resolution T1 images (3DT1). The conventional sequence was used to exclude patients with organic lesions, and BOLD-fMRI images were acquired using the Echo-Planar Imaging Gradient Echo Sequence (EPI) with the following scanning parameters: TR = 2000 ms, TE = 30 ms, scanning field = 24.0 cm × 24.0 cm, scanning matrix = 64 × 64, flip angle = 90°, layer thickness/layer spacing = 5.0/0.0 mm, voxel size = 3.75 × 3.75 × 5.00 mm^3^. In total, 32 layers were scanned and a total of 140 whole-brain images were acquired. The rs-fMRI images were scanned by instructing the participants to lie down on the scanning bed in the supine position, keep their head and body still, close their eyes, and keep their minds awake without deliberate thinking. Earplugs were provided to reduce noise, and the head was immobilized with a sponge cushion.

#### 4.5.2 Image preprocessing

SPM software (version 12.0^1^) was used to preprocess the rs-fMRI images on the MATLAB 2013b platform. First, the original images in DICOM format were converted to 3D NIFTI hdr/img format using the dcm2niigui software. Considering the uniformity of the magnetic field and adaptability of the subjects, the first five time-point images were excluded. Subsequently, image data preprocessing commenced as follows: (i) Time correction: one whole brain scan was treated as a time point, the acquisition time of the middle layer was selected as the reference time point, and the data of each time point were corrected for the time difference. (ii) Head movement correction: The head movement parameters during the scanning process of the subjects were acquired and images with excessive head movement (translation > 2 mm or rotation > 1°) were eliminated. (iii) Spatial standardization: To eliminate anatomical differences between subjects, the images of all subjects were standardized using the Montreal Neurological Institute (MNI) spatial coordinate system. (iv) Image smoothing: The images were spatially smoothed using a Gaussian kernel with a half-height full width (FWHM) of 8 mm × 8 mm × 8 mm to improve the signal-to-noise ratio of the images and remove the effect of noise on image quality.

## 5 Data collection

Data was collected using the questionnaire survey method. The participants were divided into groups: the observation group and the control group. An Enterprise Weibo group was set up. a group owner and an administrator were joined in both the observation and control groups, the group owner and administrator for questionnaire two-dimensional code distribution and data collection, The Core Self-Evaluations Scale, the PHQ-9, and GAD-7 questionnaires were administered to all participants before and at the end of the intervention, respectively, through Questionnaire Star. All the questions were mandatory, and each device can fill out a scale just once, before the intervention, and at the end of the intervention, respectively. The magnetic resonance imaging examination was carried out in two groups of 30 cases in two groups voluntarily.

Quality control was conducted before the formal intervention. Before the study commencement, the purpose and significance of the study were explained to the nurses. The principles of voluntariness and confidentiality were emphasized, and informed consent was obtained from all the participants. The research team was experienced in teaching administrators and psychologists, and during the data collation stage, double checking was used to ensure the accuracy of the data.

## 6 Statistical analysis

SPSS 20.0 statistical software was used for statistical analysis. Means, standard deviations, frequency, and constitutive ratios were used as descriptive statistics for the general information, self-evaluation, anxiety, and depression scores of the trained nurses. Independent sample t-tests were used to compare the differences in anxiety, depression, and core self-assessment scores between the two groups of trained nurses before and after the interventions. Resting-state fMRI data were used to calculate correlation coefficients between time points in brain regions, and functional network connections were used to study the changes in the brain caused by positive psychological intervention. Pearson correlation analysis was performed to analyze the change in scores measured by the nurses who voluntarily underwent magnetic resonance examination after the implementation of the intervention and functional brain imaging.

### 6.1 Calculation of neural activity indicators

First, the spatially processed rs-fMRI was delinearized using the REST 1.8 software package to remove the low-frequency drift due to the instability of the scanning hardware and thermal noise caused by spontaneous physiological activities. Subsequently, the signal was filtered by a bandpass filter of 0.01–0.08 Hz to extract the frequency signals related to neural activities. Finally, the Amplitude of Low Frequency Fluctuation (ALFF), Fractional Low Frequency Fluctuation (fALFF), and Regional Homogeneity (Reho) of each voxel were calculated from the time-domain preprocessed images to obtain whole-brain ALFF images, Reho images, normalized mean ALFF images (mALFF), mean fALFF images (mfALFF), sReho image values, and smoothed normalized Reho images (smReho) for each participant.

### 6.2 Comparative analysis of imaging metrics

SPM 12.0 software was used to compare and analyze the mALFF, mfALFF, and smReho images of the two groups of participants before and after treatment. The imaging indices of the two groups before and after treatment were statistically analyzed using two independent sample t-tests. Statistical significance was set at *p* < 0.05. All statistical results were corrected by using alphasim.

## 7 Results

### 7.1 Comparison of general information

Before intervention commencement, 65 participants were enrolled in each group. Finally, 61 participants were enrolled in the observation group (1 participant left the study and 3 participants did not complete the intervention), with 8 participants completing the magnetic resonance examination (3 participants featured failed images and 19 participants had incomplete examinations). Similarly, 58 participants were enrolled in the control group (7 participants withdrew from the study midway), and 8 participants completed the magnetic resonance examination (5 participants featured failed images and 17 participants had incomplete examinations). There were no statistically significant differences in gender, education, title, marital status, residence, nursing experience (year) and family health status (see Table 1).

**Table 1:** Comparison of general information between the two groups [participants (percentage, %)].

Characteristic		Control group (*n* = 58)	Observation group (*n* = 61)	*X*^2^ value	*p*-value
Type	Social training	44	40	0.144	0.705
	Observation	17	18		
Department	General medicine	21	19	0.509	0.992
	Neurosurgery	12	13		
	Acute and critical illness	16	14		
	Department of gynecology and obstetrics	5	5		
	Gynecology	5	4		
	Other	2	3		
Gender	Male	4	1	1.725	0.189
	Female	57	57		
Education	Specialty	42	40	<0.001	0.989
	Undergraduate	19	18		
Title	Nurses	58	56	0.160	0.690
	Nurse Practitioners	3	2		
Marital status	Married	0	2	2.139	0.144
	Singleton	61	56		
Residence	live alone	42	43	0.436	0.804
	living with parents	18	14		
	living with parents and children	1	1		
Nursing experience (years)	0–1	23	20	0.134	0.715
	1–2	38	38		
Whether family members have health problems	Yes	52	50	0.022	0.881
	No	9	8		

### 7.2 CSES

There was no significant difference between the CSES scores of the two groups before the intervention. The CSES score of the observation group was significantly higher than that of the control group post-intervention (See Table 2 for details).

**Table 2:** Comparison of CSES scores between the two groups pre- and post- intervention (score, $\bar{X}\pm S$).

Groups	Pre-intervention		Post-intervention	
	Control group (*n* = 58)	Observation group (*n* = 61)	Control group (*n* = 58)	Observation group (*n* = 61)
CSES	28.50 ± 4.52	29.59 ± 3.73	28.74 ± 2.59	35.30 ± 3.36
*t*-value	1.4377		11.885	
*p*-value	0.153		<0.001	

### 7.3 PHQ-9

There was no significant difference in PHQ-9 scores between the two groups before the intervention. The PHQ-9 scores of the observation group were significantly lower than those of the control group post-intervention. The results indicating statistically significant dimensions are presented in Table 3.

**Table 3:** Comparison of PHQ-9 scores between the two groups pre- and post-intervention (score, $\bar{X}\pm S$).

Groups	Pre-intervention		Post-intervention	
	Control group (*n* = 58)	Observation group (*n* = 61)	Control group (*n* = 58)	Observation group (*n* = 61)
PHQ-9	4.74 ± 3.32	5.02 ± 4.21	4.33 ± 3.62	3.05 ± 3.19
*t-*value	0.4015		2.0489	
*p*-value	0.689		0.043	

### 7.4 Gad-7

There was no significant difference between the GAD-7 scores of the two groups before the intervention. The GAD-7 scores of the observation group were significantly lower than those of the control group post-intervention, as detailed in Table 4.

**Table 4:** Comparison of GAD-7 scores between the two groups pre- and post-intervention (score, $\bar{X}\pm S$).

Groups	Pre-intervention		Post-intervention	
	Control group (*n* = 58)	Observation group (*n* = 61)	Control group (*n* = 58)	Observation group (*n* = 61)
GAD-7	4.25 ± 3.08	3.84 ± 3.08	4.32 ± 3.20	2.85 ± 3.15
*t*-value	0.717		2.537	
*p*-value	0.475		0.012	

### 7.5 Post-intervention comparison results of the amplitude of low frequency fluctuation

**Figure 1:**
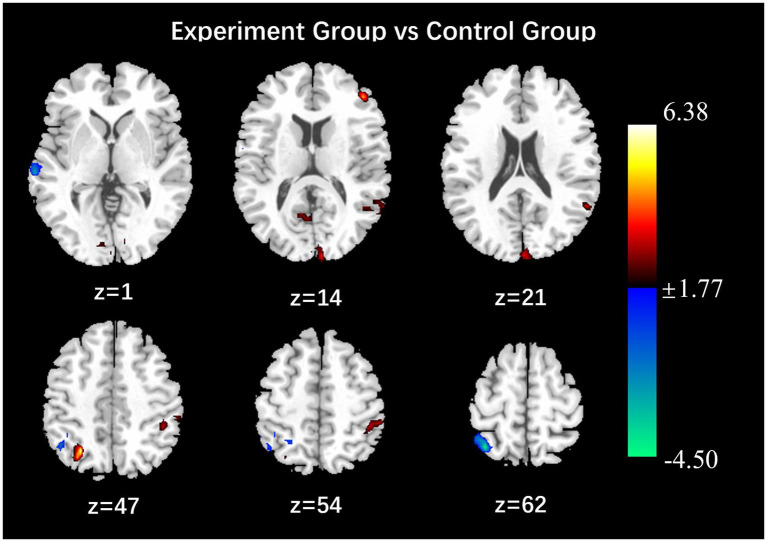
Results of comparison of ALFF values between observation and control groups (*P* < 0.05, Alphasim correction, Cluster size > 45).

**Figure 2:**
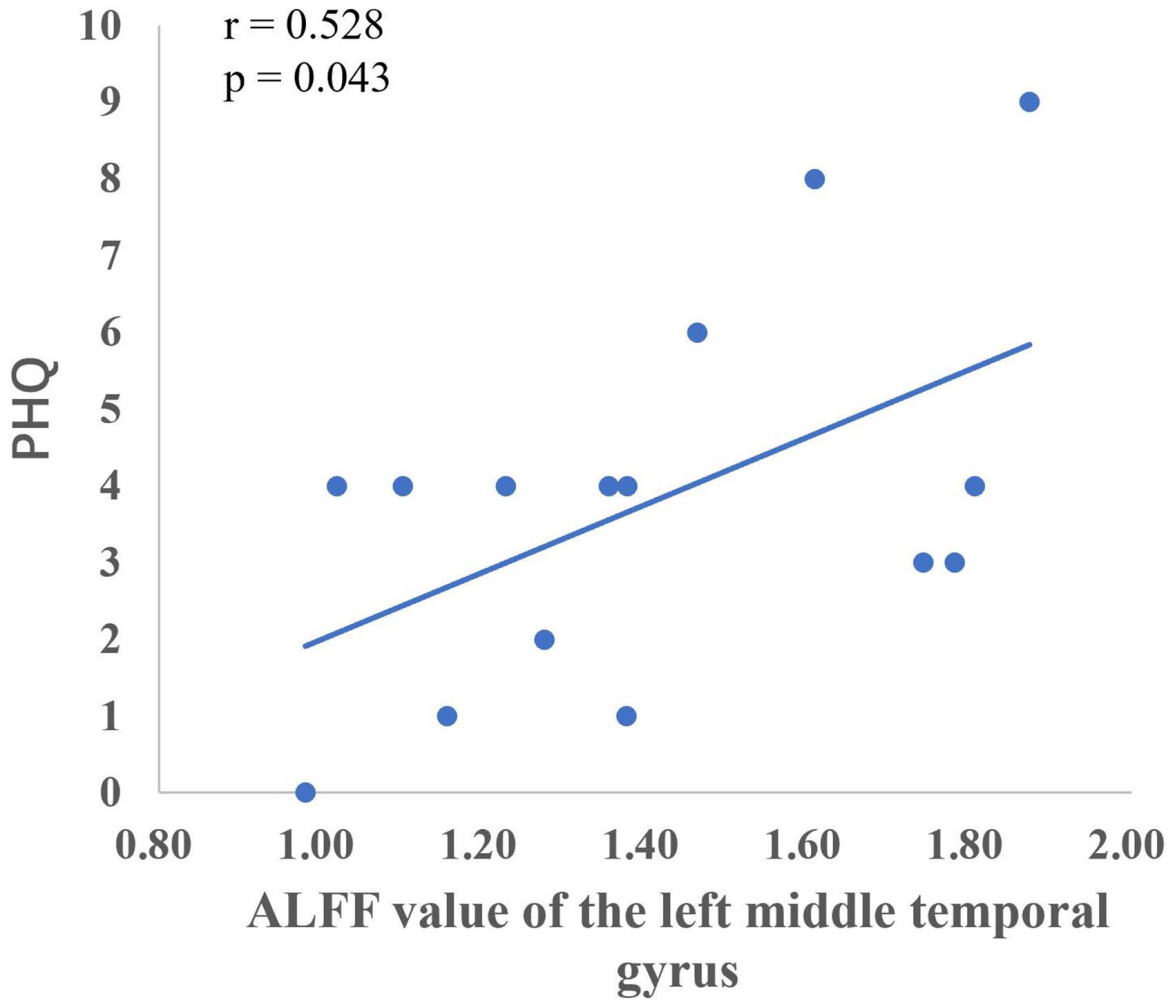
Correlation between the ALFF values of the left middle temporal gyrus and PHQ scores.

After the intervention, the group comparison showed that the ALFF values of the observation group were significantly higher than those of the control group in the bilateral calcarine gyrus, left cuneus lobe, left middle temporal gyrus, left inferior frontal gyrus, right parietal lobe, left inferior parietal lobe, right angular gyrus, and left supramarginal gyrus (see Figure 1; Table 5). After the intervention, the ALFF of the left middle temporal gyrus was significantly and positively correlated with the PHQ score (Figure 2).

**Table 5:** Differential brain region information of ALFF comparison results between the two groups post-intervention.

Brain area	Cluster size (voxels)	MNI coordinates	Peak intensity
Right parietal lobe	60	27, −63, 45	6.38
Right angular gyrus	34	33, −50, 35	3.39
Left calcarine gyrus	59	−3, −96, 24	4.12
Left inferior parietal lobe	92	−63, −33, 39	3.78
Left middle temporal gyrus	44	−57, −54, 18	3.31

### 7.6 Post-intervention comparison results of the fractional amplitude of low frequency fluctuation

**Figure 3:**
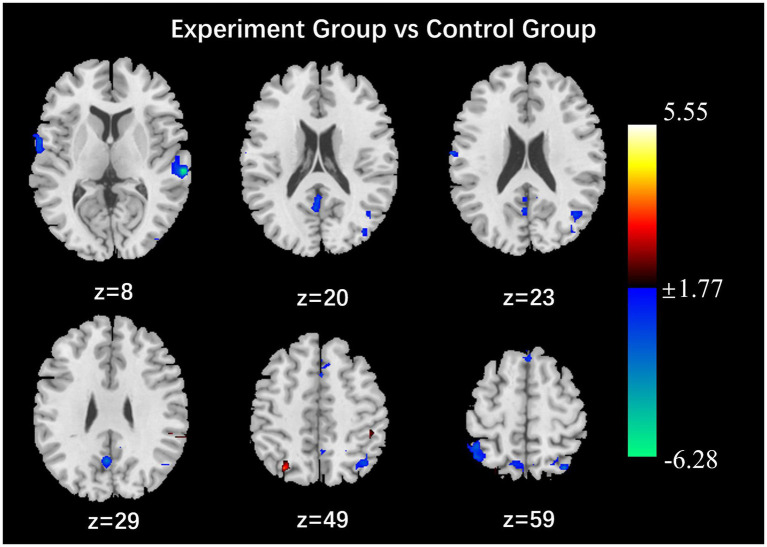
Results of the comparison of fALFF values between the observation and control groups (*p* < 0.05, Alphasim correction, Cluster size > 48).

**Figure 4:**
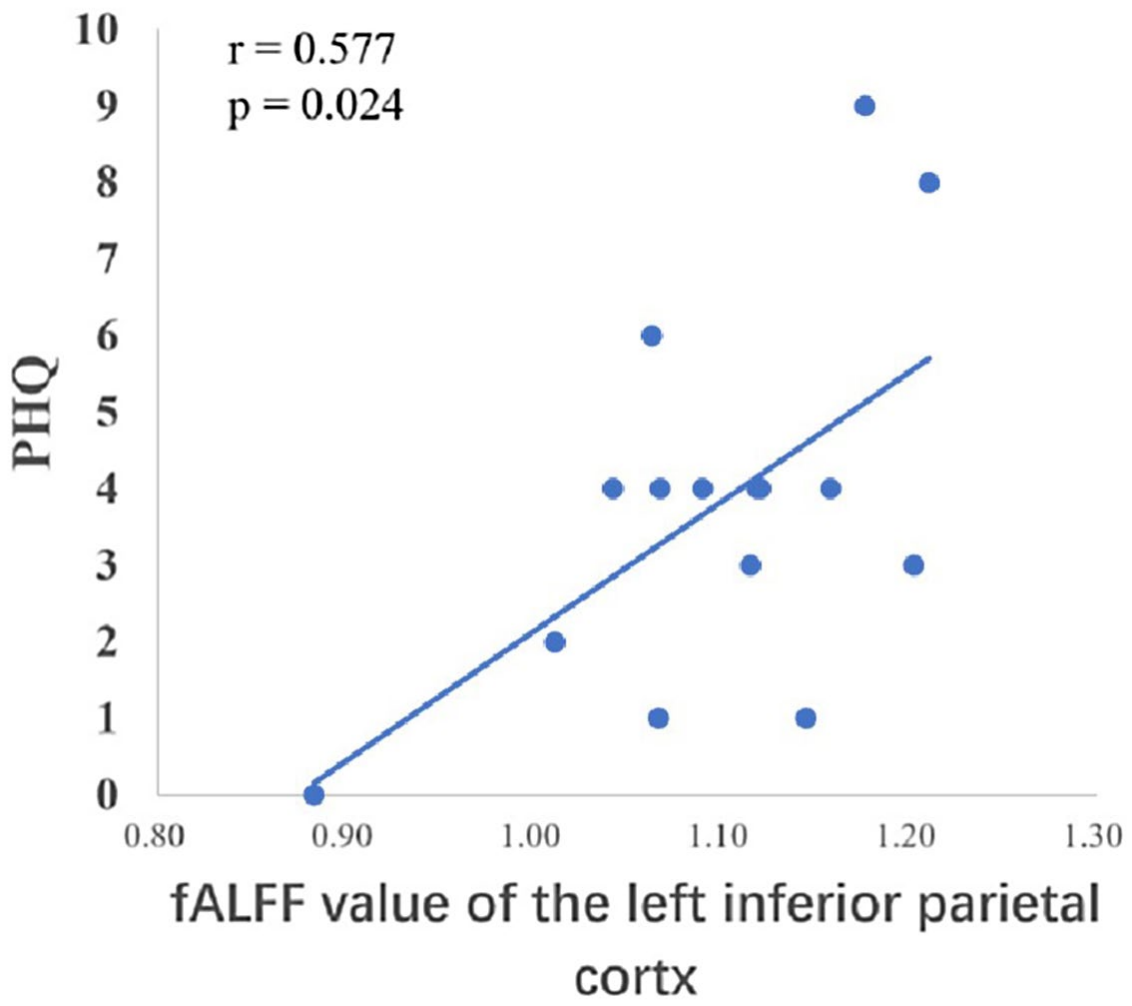
Correlation between the fALFF values of the left inferior parietal lobe and PHQ scores.

**Figure 5:**
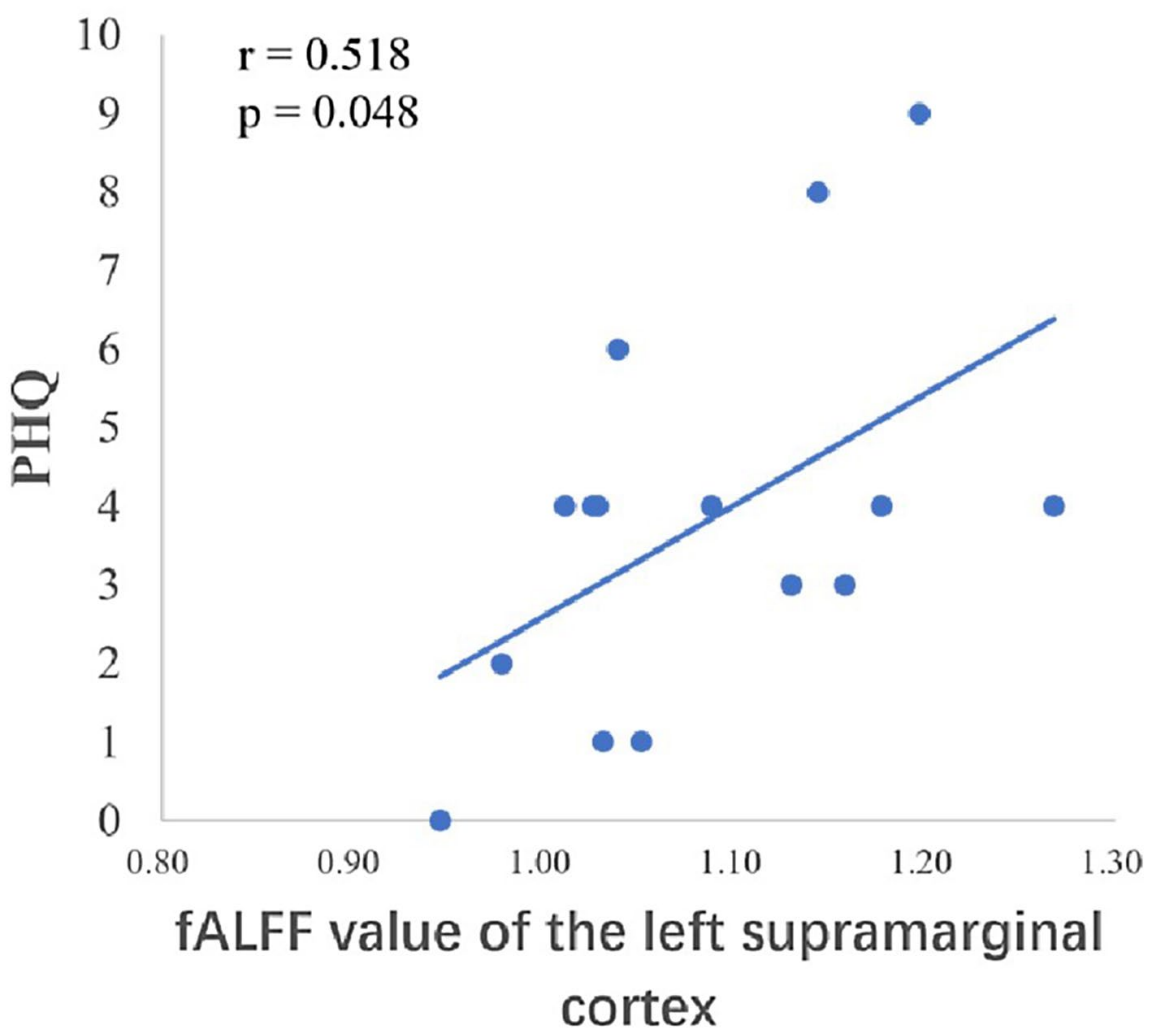
Correlation between the fALFF values of the left supramarginal gyrus and PHQ scores.

After the intervention, the group comparison showed that the observation group had significantly higher fALFF values in the right superior parietal lobe, right angular gyrus, and right middle temporal gyrus than the control group post-intervention (see Figure 3; Table 6). The fALFF values of the left inferior parietal lobe and left supramarginal gyrus were significantly and positively correlated with PHQ scores (Figures 4, 5).

**Table 6:** Differential brain region information for the results of fALFF comparison between the two groups post-intervention.

Brain area	Cluster size (voxels)	MNI coordinates	Peak intensity
Right parietal lobe	56	30, −69, 48	4.05
Right angular gyrus	25	33, −50, 36	3.18
Left orbital middle frontal gyrus	35	−15, 60, −12	3.73
Left inferior parietal lobe	59	−60, −33, 36	2.89
Left supramarginal gyrus	25	−54, −42, 30	2.71

### 7.7 Post-intervention comparison results of the regional homogeneity

**Figure 6:**
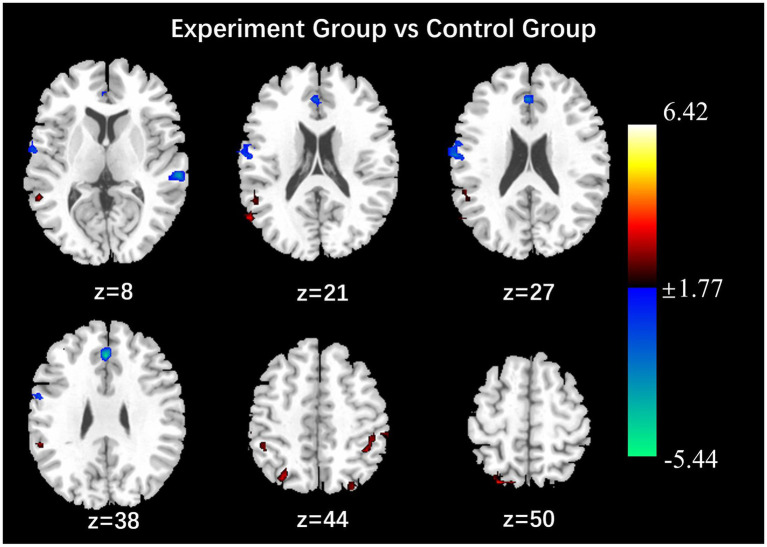
Results of the comparison of Reho values between the observation and control groups (*P* < 0.05, Alphasim correction, Cluster size > 50).

**Figure 7:**
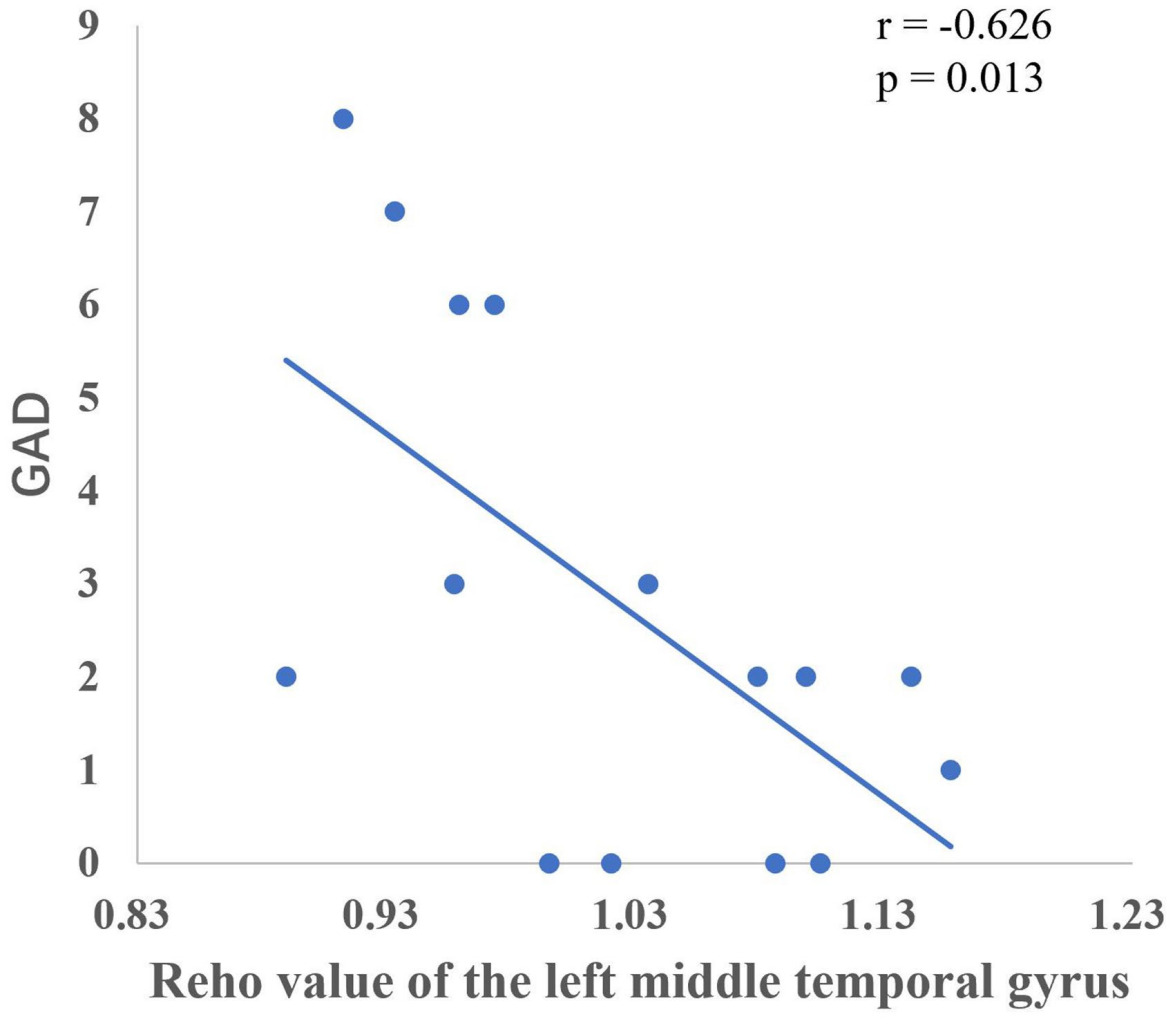
Correlation between the Reho values of the left middle temporal gyrus and GAD scores.

The group comparison showed that the observation group had significantly higher Reho values in the bilateral superior parietal lobe, bilateral supramarginal gyrus, and bilateral inferior parietal lobe than the control group post-intervention. The results are shown in Figure 6 and Table 7. Reho values in the left middle temporal gyrus were significantly negatively correlated with GAD scores (Figure 7).

**Table 7:** Differences in Reho value comparison results between the two groups post- intervention brain area information.

Brain area	Cluster size (voxels)	MNI coordinates	Peak intensity
Right middle frontal gyrus	44	27, 54, 39	6.42
Right parietal lobe	46	36, −60, 36	5.13
Right angular gyrus	42	30, −50, 42	4.16
Right supramarginal gyrus	44	54, −36, 42	4.83
Right inferior parietal lobe	34	45, −39, 42	4.51
Left inferior parietal lobe	97	−63, −33, 39	4.14
Right middle temporal gyrus	45	57, −66, 18	3.35
Left middle temporal gyrus	39	−51, −21, −12	3.22

## 8 Discussion

### 8.1 Documenting “Three Good Things” can be effective in enhancing nurses’ core self-evaluations and improving the psychological state of nurses in training during the transition period

Positive psychology is an effective way to mobilize the positive forces of nurses, exert positive initiative, improve work efficiency by tapping into their own strengths and look at dealing with problems in a positive light to enhance the subjective well-being of nurses (Wu, 2024; Seligman et al., 2005). According to a previous study (Xiao et al., 2020), new nurses are prone to serious negative psychological emotions during standardized training. “Three Good Things” of the positive psychology can effectively reduce the work pressure on people and improve their psychological state (Cline et al., 2022; Schienle et al., 2025). In a survey of 486 healthcare workers, Rippstein-Leuenberger et al. (2017) found that the “Three Good Things” could help healthcare workers develop positive emotions, reduce anxiety and depression, and enhance their sense of well-being. Sexton and Adair (2019) showed that the “Three Good Things” could help healthcare workers reduce stress and negative emotions, and experience pleasant emotions. Gold et al. (2023) found that the observation group showed a significant reduction in negative emotions after 12 weeks of intervention based on “Three Good Things”. In this study, firstly, lectures and interactions were used to help nurses recognize and understand positive psychology, and then nurses in training were urged and guided to discover the good things in their life and work, clock in, and record three good things at regular intervals, and were given inspirational questions to reflect on the reasons for the “Three Good Things,” which is conducive to the nurses’ discovery of their own value, improvement of their self-centeredness, alleviation of anxiety, depression and other negative emotions brought about by the transition period, and the creation of a healthy and positive psychological state. The results of this study showed that both groups reported lower CSES scores and higher PHQ-9 and GAD-7 scores pre-intervention. Twelve weeks post-intervention, the CSES scores of the observation group were significantly higher than those of the control group, and the PHQ-9 scores and GAD-7 scores were significantly lower than those of the control group, which suggests that the positive psychological intervention based on “Three Good Things” effectively improved the psychological state of trained nurses during the transition period.

### 8.2 Documenting “Three Good Things” can improve nurses’ perceptions of their work life

The ALFF reflects changes in the intensity of local spontaneous neural activity in the brain, and the ALFF value is directly proportional to the intensity of neuronal activity in brain regions. The fALFF reflects the resting-state brain spontaneous neural activity, and its analysis aims to reveal the characteristics of autonomous activity in different brain regions. The Reho reflects the synchronization of whole-brain voxels during local regional brain functional activities, and the higher the Reho value, the higher the consistency and centrality of regional brain activities. The higher the Reho value, the higher the coherence and centrality of regional brain activities (Yang et al., 2022; Yu et al., 2021; Wu et al., 2024).

The frontal lobe, cuneus, middle temporal gyrus, inferior frontal gyrus, angular gyrus, calcarine gyrus and supramarginal gyrus have been identified as key centers for processing visual, spatial and emotional information. These regions have been shown to play a primary role in regulating the brain’s cognitive, emotional, memory, attention, executive function, and other processes (Briggs et al., 2019; Briggs et al., 2021; Xie et al., 2023; Li et al., 2022). Cuneate and middle temporal gyrus low-frequency amplitude values are reduced, and judgment of visual images and spatial relationships is reduced, parietal cortex activity and attention will be reduced (Xu et al., 2023). Previous studies have shown that depressed patients have reduced ALFF values in the bilateral medial superior frontal gyrus and the bilateral precuneus (Zhou et al., 2023), ALFF values in the right and left superior frontal gyrus, middle frontal gyrus, and right inferior frontal gyrus brain regions were elevated during the reception of positive emotional stimuli (Chen et al., 2011). The results of this study in the observation group are as follows: the ALFF values of the bilateral calcarine gyrus, left lateral cuneate lobe, left medial temporal gyrus, left inferior frontal gyrus, right superior parietal lobe, left inferior parietal lobe, right angular gyrus, and left supramarginal gyrus were higher than those of the control group. It was observed that nurses in the observation group who received the positive psychological intervention exhibited low-frequency amplitude (ALFF) in multiple brain regions that are closely associated with cognitive and emotional processing functions following the intervention. This finding suggests that the positive psychological intervention may enhance individuals’ emotion management abilities by modulating neural activity in brain regions implicated in emotional processing. The fALFF values of the right superior parietal lobe, right angular gyrus, and right middle temporal gyrus were higher than those of the control group, and the Reho values of the bilateral superior parietal lobe, bilateral superior marginal gyrus, and bilateral inferior parietal lobe were higher than those of the control group. It was noted that depressed patients had reduced Reho values in the left inferior temporal gyrus, right middle temporal gyrus, and superior frontal gyrus, and significantly increased fALFF values in the left middle temporal gyrus (Li et al., 2022; Zhou et al., 2022; Zhao et al., 2024). This suggests that the intervention may improve the neural basis of emotion regulation by enhancing prefrontal-limbic system functional connectivity.

The temporal lobe is primarily responsible for perceptual information processing, and the middle temporal gyrus is mainly involved in functions such as distance discrimination, familiar face recognition, and sentence comprehension (Catani, 2022). The results of the Pearson correlation analysis showed that the ALFF value of the left middle temporal gyrus was positively correlated with PHQ scores, and the Reho value of the left middle temporal gyrus was significantly negatively correlated with GAD scores, which may be related to changes in the departmental environment and personnel during the rotation period of nurses in standardized training. Li et al. (2019) found that new nurses rotated through one department every 6 months when they entered the standardized training stage, and with changes in the department environment, personnel, and specialty diseases, nurses had no sense of belonging, which could easily cause depression. The correlation between the fALFF values and PHQ scores of the left inferior parietal lobe and left supramarginal gyrus in this study suggests that these regions play key roles in the reception and processing of emotional cognition.

However, this study has some limitations. Firstly, the study participants were new nurses in a hospital individuals from Nanchong (a prefecture-level city in southwestern China), and the psychological state of nurses in transition was not discussed in relation to their demographic factors, such as gender, marital status, economic income, and family background. Due to differences in socio-cultural and economic development, some findings may differ from other countries. It is recommended that this relationship needs to be explored in a follow-up study. Secondly, the study’s sample size was relatively limited, particularly the number of participants who underwent MRI examinations. The number of MRI examiners is decreasing due to factors such as people leaving their jobs, refusing to have an MRI because of the closed environment (claustrophobia) or fear of the effects of radiation from the exam on their health, dropping out of the exam, being unable to have an MRI because of metal implants in the body, and scheduling conflicts. Further multi-center, large-scale intervention studies are needed for a more general interpretation of MRI results in the future. Thirdly, routine training was used as the control group, categorized as ‘routine practice control’. However, as no active control group was employed, the potential influence of non-specific factors, such as routine training, on the effectiveness of the ‘Three Good Things’ intervention could not be completely excluded. In subsequent studies, the addition of an active control group is intended to enhance the experimental design and ensure the reliability of the findings. Despite these limitations, we believe that this study is valuable as it enhances our understanding of the “Three Good Things”. The selected study population comprised new nurses in the hospital.

In summary, positive psychological interventions may regulate nurses’ psychological states by enhancing neural activity in brain regions associated with emotion regulation. The “Three Good Things” have been shown to assist nurses in training during the transition period to enhance their core self-assessment ability, alleviate their anxiety and depression, and improve their sense of occupational identity and mental health. Furthermore, the “three good things” provide a theoretical basis for nursing administrators to formulate a teaching programme for nurses in training.

## Data Availability

The original contributions presented in the study are included in the article/supplementary material, further inquiries can be directed to the corresponding author.

## Footnotes

^1^ http://www.fil.ion.ucl.ac.uk
